# Adherence to Self-Care Recommendations and Associated Factors among Adult Heart Failure Patients in West Gojjam Zone Public Hospitals, Northwest Ethiopia

**DOI:** 10.1155/2022/9673653

**Published:** 2022-12-21

**Authors:** Belayneh Molla, Haimanot Abebe Geletie, Girma Alem, Tenaw Gualu, Bitew Tefera Zewudie, Shegaw Tesfa, Tadesse Tsehay, Baye Tsegaye Amlak

**Affiliations:** ^1^Department of Nursing, Hailu Alemu College, Finote Selam, Ethiopia; ^2^Department of Nursing, Wolkite University, Wolkite, Ethiopia; ^3^Department of Nursing, Debre Markos University, Debre Markos, Ethiopia

## Abstract

**Background:**

Self-care practices are an important part of heart failure patient management and essential to control symptoms of the disease and its exacerbation. However, poor adherence to these self-care behaviors could be associated with an increase in hospitalization, morbidity, and mortality. Even if it is an important part of management for heart failure patients, yet information is not adequate in the study area about adherence to self-care recommendations and associated factors among heart failure patients.

**Purpose:**

To assess self-care recommendation adherence and associated factors among heart failure patients in West Gojjam Zone public hospitals.

**Methods:**

Institutional-based cross-sectional study was conducted on 304 selected heart failure patients attending follow-up at public hospitals in West Gojjam Zone from March 16 to April 16, 2021. Consecutive sampling technique based on patient arrival with proportional allocation to each hospital was employed to select the study participants. Data were collected through face-to-face interview and reviewing patients' medical records. Data were entered into EpiData version 3.1 and analyzed using Statistical Package for Social Sciences (SPSS) version 25. Binary logistic regression model was fitted to assess the association between adherence to self-care recommendations and associated factors. *P* value < 0.05 with 95% confidence interval (CI) was considered to declare a statistically significant association in multivariable logistic regression.

**Results:**

In this study, 304 patients participated with a response rate of 97.4%. Only 32.9% of them had good adherence to self-care recommendations. Having good knowledge on heart failure (adjusted odds ratio (AOR) = 4.6; 95% CI: 1.82, 11.86), no depression (AOR = 6.1; 95% CI: 1.92, 19.37), having strong social support (AOR = 3.57; 95% CI: 1.56–8.33), age 30-49 years (AOR = 3.37; 95% CI: 1.14, 9.89), and college and above level of education (AOR = 6.17; 95% CI: 1.22, 31.25) were factors significantly associated with good adherence to self-care recommendations.

**Conclusion:**

This study showed that most of the heart failure patients had poor adherence to self-care recommendations. Policymakers and other stakeholders should develop and implement appropriate strategies to increase patients' adherence level to self-care recommendations by emphasizing on addressing identified factors.

## 1. Introduction

Heart failure (HF) is a clinical syndrome characterized by signs and symptoms of fluid overload or inadequate tissue perfusion due to decreased pumping activity of the heart [1]. It is a major public health concern and leading cause of death worldwide [2]. It is one of the predominant chronic diseases affecting 37.7 million people worldwide [3]. The global mortality rate of patients with heart failure was about 16.5%, highest in Africa and India [4]. Despite significant advances in therapies and preventions, mortality and morbidity are still high, which significantly affects quality of life [5].

Heart failure is managed both pharmacologically and nonpharmacologically by applying self-care recommendations (SCR), and treatment begins with providing comprehensive education and counseling to the patients and families [1]. Despite advances in pharmacological therapy, HF morbidity and mortality remain high and more emphasis should be placed on nonpharmacological management of HF whose main aspect is self-care management [6]. Self-care recommendations are activities that heart failure patients must undertake to care for himself/herself to promote health and well-being, which is an important part of heart failure management [7]. Self-care activities for heart failure clients include taking prescribed medications as ordered, following dietary and fluid restriction, engaging in exercise, monitoring and recognizing early symptoms, seeking appropriate medical assistance, and modifying their self-concept [8]. Monitoring weight on a regular basis, respond to a sudden unexpected weight gain, restrict fluid to 1.5–2 L/day, assess and report symptoms like breathlessness and fatigue, need of advice and help to stop smoking, limit daily alcohol intake, engage in regular moderate daily activity for at least 30 min, and vaccination against pneumococcal disease are recommended for heart failure patients [9]. Interventions that can lead to sustained improvement in patients' adherence to treatment recommendations can be achieved with the aid of coordinated measures such as patients' education and regular follow-up contacts [10].

Regardless of the recommendations, most studies showed that heart failure patients had low adherence to self-care recommendations. A study in the American College of Cardiology showed that adherence to individual recommendations ranged from 89% for medication to 26% for exercise [11]. Also, in Africa including Ethiopia, most heart failure patients had low adherence to high-sodium diet restriction, regular exercise, weight monitoring, and fluid intake restriction [12–14]. A cross-sectional assessment of self-care behaviors in some regions of Ethiopia found that between 54.2 and 72% of heart failure patients have poor adherence to self-care recommendations [15–18]. Some of the previously conducted studies showed that factors associated with adherence to self-care recommendations among heart failure patients were gender [19], age [20], functional status and social support [21], knowledge [22], monthly income [23], duration of HF and comorbidity [24], New York Heart Association (NYHA) functional classification [13], and psychological attributes [25].

Poor adherence to SCRs may result in an increased risk of morbidity and mortality, diminished quality of life, and increased health care costs associated with increased outpatient care and increased rates of hospital readmission [26–30]. Approximately 1/4 of patients discharged after treatment for HF are readmitted to the hospital within 30 days [31], due to failing to follow the therapeutic recommendations [32].

Even though there is an increasing incidence of HF-related morbidity and mortality in Africa, including Ethiopia [33, 34], evidence regarding HF patients' adherence to self-care recommendations was limited and some of the studies conducted previously revealed different findings on the level of adherence to self-care recommendation and that there might be different findings on this study area [15–18]. This study was also conducted at zonal level encompassing seven hospitals, but others were at a specific health institution; the results of other studies vary in finding that conclusion cannot be made to this study area and these few available evidences are inadequate for generalization, and some of the other studies also differ in study population as they include all age groups. This assessment of heart failure patients' adherence to self-care recommendations in this area is a key for the prevention of disease worsening and improved quality of patients' lives. Therefore, this study is aimed at assessing the level of adherence to self-care recommendations and associated factors among heart failure patients in West Gojjam Zone public hospitals, Northwest Ethiopia.

## 2. Materials and Methods

### 2.1. Study Design and Setting

An institutional-based cross-sectional study was conducted at 7 public hospitals of West Gojjam one, Amhara Region of Ethiopia, from March 16 to April 16, 2021. West Gojjam is one of the administrative zones in Amhara Regional State, Ethiopia, with the capital city of Finote Selam, which is located 387 km away from Addis Ababa, capital city of Ethiopia, and 176 km away from Bahir Dar, capital city of Amhara Regional State. The total population of the zone was 2,106,596 in 2019; among this, 1,058,272 were men and 1,048,324 were female [35]. There were seven primary public hospitals in this zone, namely, Finote Selam, Burie, Durbete, Merawi, Adet, Liben, and Feres Bet. All public hospitals found in West Gojjam Zone provide health care services for the catchment population both in inpatient and outpatient settings, and the total number of adult heart failure patients on follow-up in those hospitals was 465. Previously, diagnosed heart failure patients who were 18 years old or above, having at least one-month follow-up prior to data collection period, were included in this study.

### 2.2. Sample Size and Sampling Methods

**Figure 1:**
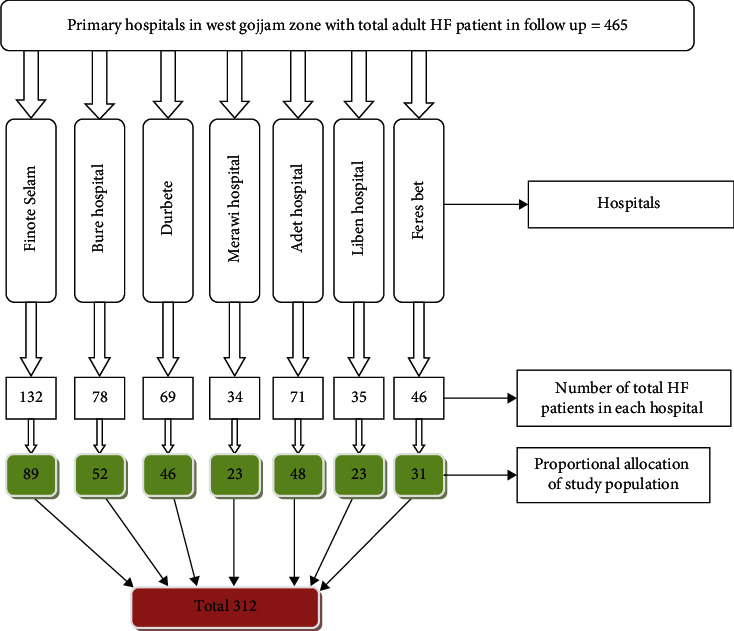
Sampling procedure to determine adherence to self-care recommendations and associated factors among heart failure patients in West Gojjam Zone public hospitals, 2021.

The minimum required sample size for this study was calculated using both single and double population proportion formulas by taking the proportion of patients' adherence to self-care recommendations as 22.3% based on the study conducted in Western Ethiopia [19], at 95% confidence interval (CI) by assuming a margin of error 5% = 0.05, 80% power, and 1 : 1 ratio of exposed to nonexposed outcomes and adding 10% for possible nonresponse rate, and taking the maximum value; the final sample was 312 based on the above assumptions. There were 7 public hospitals in West Gojjam Zone with a total of 465 adult heart failure patients, namely, Finote Selam Primary Hospital (132), Bure Primary Hospital (78), Durbete Primary Hospital (69), Merawi Primary Hospital (34), Adet Primary Hospital (71), Liben Primary Hospital (35), and Feres Bet Primary Hospital (46). Consecutive sampling technique based on patient arrival with proportional allocation to each hospital was employed to select the study participants (Figure 1).

### 2.3. Operational Definitions

- Poor adherence: patients who scored <75 on self-care recommendation Likert scale questions [13, 19, 22]
- Poor knowledge: heart failure patients who answered lower than 75% of heart failure knowledge questions correctly [13, 19, 36]
- No depression: those who scored <10 of total depression questions [37]
- Poor social support: Oslo Social Support (OSS-3) item sum scores 3–8, moderate social support: OSS-3 item sum scores 9–11, and strong social support: OSS-3 item sum scores 12–14 [38]

### 2.4. Data Collection Tool and Procedure

Pretested semistructured questionnaire developed after the review of similar literatures and standardized tools for level of adherence were used to collect data. Face-to-face interview with medical chart review was employed to obtain data from the selected participants. The tool consists of five parts: these are sociodemographic and patient profiles, HF knowledge, depression, social support, and heart failure self-care behaviors.

Adherence to self-care recommendations was measured by using the Revised Heart Failure Self-Care Behavior Scale [19]. The Revised Heart Failure Self-Care Behavior Scale contains 26 items grouped into five components of Orem's self-care requisites: these are seeking and securing appropriate medical assistance for their HF; being aware of and attending to the effects and results of HF; effectively carrying out medically prescribed diagnostic, therapeutic, and rehabilitative measures directed towards the prevention of exacerbations or complications of HF; modifying the self-concept in accepting oneself as having HF; and learning to live with the effects of HF and its treatments [39]. Participants were asked to score how often they applied each recommendation. Adherence was measured by the 6-point Likert scale (where 0 = none of the time, 1 = a little of the time, 2 = some of the time, 3 = a good bit of the time, 4 = most of the time, and 5 = all of the time). Patients were categorized as adherent if they had a score of 75 and above, which corresponded to being adherent most of the time or all of the time [8].

The Japanese heart failure knowledge scale was used to assess patients' knowledge of heart failure. Out of 15 questions, four questions were about HF signs and symptoms, nine questions about HF self-care recommendations, and two general questions. Hot bath item was modified based on rural versus urban in which hot bath using water heater operated in the presence of electricity is practiced in urban areas and hot bath by heating water using other source of energy like wood fuels and charcoal is practiced in rural areas in Ethiopia. For knowledge questions, one point was given for each correct answer; zero point was given for incorrect and “I don't know” responses. Based on this, the minimum and maximum possible summation scores were 0 and 15, respectively, and those patients who answered greater than or equal to 75% of knowledge questions correctly were considered as having good knowledge, and others were considered as having poor knowledge [36].

Depression was measured by using the Patient Health Questionnaire (PHQ-9) for depressive symptoms, which ranges from 0 to 27 scores. The PHQ-9 tool contained nine questions, each having four options (0 = not at all, 1 = several days, 2 = more than half of the days, and 3 = nearly every day) which were used to screen depression from the study participants [37]. The Oslo Social Support Scale consisting of three items was used for assessing the level of social support. The sum score ranges from 3 to 14, with high values representing strong levels and low values representing poor levels of social support [38]. Data were collected in alignment with the study participants' follow-up date by seven trained Bachelor of Science degree nurses. Patients' medical records were also reviewed for secondary data such as presence of comorbidity, NYHA functional classification, history of hospitalization, and duration of illness after disease diagnosis.

### 2.5. Data Quality Control

To ensure data quality, training was given to data collectors and supervisors by the investigators on how to collect data from patient charts and how to conduct patient interviews. Pretest was conducted in 5% of sample one week before actual data collection outside of the study area (in Yejuba Primary Hospital), and necessary modifications were made before actual data collection. The completeness of each questionnaire was checked before analysis. Simple frequencies and crosstabulation were done to identify missing data and inconsistency. Cronbach's alpha value was used to check the reliability of the tool, and its quality was assured through experts' evaluation.

### 2.6. Data Processing and Analysis

Data were entered into EpiData version 3.1 and cleaned, coded, and analyzed using SPSS 25.0 [40]. For knowledge questions, correct answers were coded as “1” and incorrect and “I don't know” responses were coded as “0” and those patients who answered greater than or equal to 75% of knowledge questions correctly were considered as having good knowledge. Summation was computed for PHQ-9 for depression, and those who scored <10 point summation scores were considered as having no depression. Similarly, summation was computed for OSS-3 items and those who scored 3–8 were considered as having poor social support, and summation scores 9-11 and 12-14 were analyzed as having moderate and strong social support, respectively. Descriptive statistics were computed for sociodemographic variables, participant profiles, HF knowledge, social support, depression, and self-care recommendations. A binary logistic regression model was fitted to assess the association between adherence to self-care recommendations and independent variables. All independent variables that were associated with the dependent variable in bivariable analysis with a *P* value of 0.25 or less were included in the multivariable analysis. The crude odds ratio (COR) and adjusted odds ratio (AOR) together with their corresponding 95% CI were computed. A *P* value < 0.05 and corresponding 95% CI of odds ratio were considered to declare a factor as statistically significant, and the results of this study were presented in narration, tables, and figures. Moreover, variance inflation factor and tolerance to check for multicollinearity and Hosmer and Lemeshow goodness-of-fit test to check for model fitness were computed.

## 3. Results

### 3.1. Sociodemographic Characteristics

In this study, 304 heart failure patients participated with a response rate of 97.4%. Among the study participants, 178 (58.6%) were men. Participants' mean age was 55.7 years (±8.4 years), 110 (69.1%) were married, and 213 (70.1%) lived in rural areas. Less than half of the study participants (138 (45.4%)) were unable to read and write, and most of the study participants (286 (94.1%)) had health insurance (see Table 1).

### 3.2. Clinical Characteristics of Study Subjects

From 304 study participants, 110 (36.2%) of them have at least one chronic comorbidity in addition to heart failure, of which hypertension (HTN) (14.1%) and kidney disease (KD) (10.2%) were the most common followed by diabetes mellitus (DM). More than half of the study participants 160 (52.6%) were NYHA class III heart failure patients. Most of the study participants 266 (87.5%) were initially diagnosed as having heart failure more than one year ago, and almost half of the them 154 (50.7%) had no history of hospitalization related to heart failure for the last one year (see Table 2).

### 3.3. Participants' Knowledge on Heart Failure

Most of the study participants 262 (86.2%) had a poor level of knowledge regarding heart failure signs and symptoms, disease conditions, and self-care management of heart failure (Table 3).

### 3.4. Depression Symptoms and Level of Social Support

Among the study participants, most of them 258 (84.9%) had no depression (Table 4) and more than half of them, 181 (59.5%), had strong social support; 28.94% of them had moderate social support, and the remaining 11.5% of them had poor social support (Table 5).

### 3.5. Level of Adherence to Self-Care Recommendations

**Figure 2:**
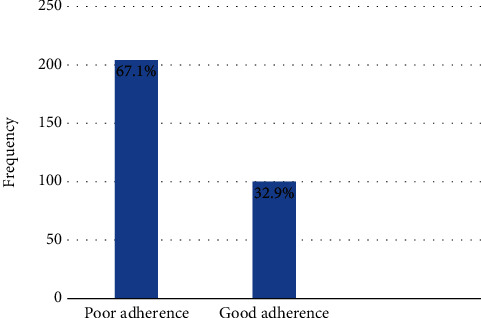
Adherence to self-care recommendations among HF patients, in West Gojjam Zone public hospitals, Northwest, Ethiopia, 2021.

From a total of 304 study participants, only 100 (32.9%) had overall good adherence to self-care recommendations (Figure 2).

### 3.6. Individual Self-Care Recommendations

From individual self-care recommendations, higher levels of good adherence were noted for not smoking, limiting alcohol intake, taking medication regimen, and appointment keeping. Least frequently performed self-care recommendations were watching how much water passes, not drinking too many fluids, and putting the feet up when sitting on a chair (see Supplemental File 1).

### 3.7. Factors Associated with Adherence to Self-Care Recommendations

In bivariate logistic regression analysis, factors associated with adherence to self-care recommendations at *P* value ≤ 0.25 were age, marital status, residence, level of education, comorbidity, NYHA classification, history of hospitalization, depression, HF knowledge, and level of social support. In multivariable logistic regression analysis, age, level of education, depression, HF knowledge, and level of social support were statistically significant at *P* value < 0.05 with 95% confidence interval (see Supplemental File 2).

The results of this study showed that those who had good knowledge of HF were nearly five times (AOR = 4.6; 95% CI: 1.82, 11.86) more likely to adhere to HF self-care recommendations than those who had poor knowledge. Patients with no depression were six times (AOR = 6.1; 95% CI: 1.92, 19.37) more likely to have good self-care adherence than those who had depression. Patients having moderate social support were 0.28 times (AOR = 0.28; 95% CI: 0.12-0.64) less likely to be adherent to self-care recommendations than those who had strong social support. Participants aged 30-49 years were found three times more likely to adhere to HF self-care recommendations than those aged ≥70 years (AOR = 3.37; 95% CI: 1.14, 9.89). Patients with primary level of education were three times (AOR = 3.22; 95% CI: 1.15, 8.99), those with high school level of education were four times (AOR = 4.17; 95% CI: 1.36, 12.76), and patients having college/university level of education were found six times (AOR = 6.17; 95% CI: 1.22, 31.25) more likely to be adherent to SCRs than those who have no formal education (Table 6).

## 4. Discussion

This study assesses the level of self-care adherence and its predictors in patients with heart failure by defining good adherence as >75 point cumulative score. Based on this study, the capability to perform self-care was less than its therapeutic self-care demands. As a result, only 100 (32.9%) of study participants with 95% CI (28%, 38.4%) had good adherence to self-care recommendations. This result is relatively similar to studies done in West Amhara Region Referral Hospitals (37.7%) [41], Atlanta (35.7%) [42], and Korea (31.9%) [21]. The possible explanation might be similar study design, population, and similarity in tool used to determine the level of adherence to self-care recommendations. The result of this study was lower as compared to studies done in Jimma University specialized hospital (40.8%) [17], Kenya (49.2%) [43], and Netherlands (72%) [44]. The low results in this study might be due to difference in setting; those studies were conducted in referral and specialized hospitals with better facilities and assistive devices for self-care practices than primary hospitals and difference in cut-point to determine level of adherence.

However, it was higher compared to the study done in Gondar University Specialized Teaching Hospital (22.3%) [19]. The high result in this study could be due to difference in sex distribution among participants that men were predominant in this study than a study conducted in Gondar, in which males were found to be more adherent than females, as evidenced in other study [45]. The other justification for this discrepancy might be a difference in setting, in which patients treated in specialized hospitals are usually debilitated, needing intensive care with comorbidities that affect their capability to adhere to self-care recommendations [46, 47].

In this study, patients aged 30-49 years were more likely to adhere to HF self-care recommendations than those who were >70 years of age. Consistent findings were shown in studies done in Jimma University Specialized Hospital [13], Atlanta [20], and a systematic review of European Heart Failure Self-Care Behavior Scale studies [44]. Having old age is associated with decreased physical ability and mental activity and dependency on others for self-care activities leading to poor compliance to recommendations [48].

Patients with higher educational levels were found to be more likely adherent to HF self-care recommendations than those who did not attend formal education. This result is in agreement with the studies conducted in Atlanta [20], Nepal [45], and a systematic review of European Heart Failure Self-Care Behavior Scale studies [44]. This can be explained by individuals with higher educational level having higher level of reasoning and decision-making for performing self-care behaviors leading to good adherence [49]. It is well known that education gives people knowledge of the world around them and changes it into something better and individuals with HF and poor literacy had difficulties in navigating the health system and understanding the information required for self-care [50].

Also, in this study, those who had good knowledge on heart failure were more likely adherent to self-care recommendations than those who had poor knowledge. A similar finding was shown in studies done in West Amhara Region Referral Hospitals, Jimma University Specialized Teaching Hospital [13], Gondar University Referral Hospital [19], Korea [21], Netherland [22], and a systematic review of European Heart Failure Self-Care Behavior Scale studies [44]. This might be explained by the fact that patients' knowledge about the disease is a prerequisite to improve self-care behaviors and avoid rehospitalizations [51]. According to Orem's self-care theory, knowledge is a power that enables self-care; it must be specific and organized around the meeting of known self-care requisites. Proper understanding of the inhibiting and promoting effects of basic conditioning factors on self-care performance helps patients meet their requirements for self-care [39]. This implies that patients who had poor self-care because of low levels of HF knowledge could require attention during counseling for effective self-care practices [52].

Patients who had no depression were more likely to have good self-care adherence than those who had depression. The result is in agreement with the studies conducted in West Amhara Region Referral Hospitals [25], Netherlands [22], and a systematic review of European Heart Failure Self-Care Behavior Scale studies [44]. Patients with better mental health are more adherent to self-care recommendations due to unaltered thinking ability and positive attitude towards health maintenance [53]. Depression can reduce self-care maintenance indirectly by decreasing self-care confidence [54]. This implies that efforts to improve self-care maintenance by managing depression in vulnerable individuals are essential, and patients with depression need additional interventions during follow-up [55].

The other finding of this study showed that patients with moderate social support were less likely to be adherent to self-care recommendations than those who had strong social support. This finding was similar with other studies conducted in West Amhara Region [25], Nepal [45], and Korea [21]. Social support might facilitate adherence to self-care through cognitive and affective mechanisms such as increasing self-efficacy of control over heart failure [56]. Support from family, friends, and health care providers can help patients monitor their symptoms and carry out healthy behaviors effectively by promoting self-care maintenance and assisting with their daily activities [57].

## 5. Conclusion

In this study, one out of every three heart failure patients reports that they have good adherence to self-care recommendations. Advanced age, low level of education, poor awareness of heart failure, depression, and decreased level of social support were predictors of poor compliance to self-care recommendations. This implies that policymakers and other stakeholders should develop and implement appropriate strategies to increase patients' adherence level to self-care recommendations by emphasizing on addressing identified factors.

### 5.1. Limitation of the Study

In this study, self-care practices were measured only by self-reporting that recall bias might have occurred, and actual data on prescribed drug regimens were not available, and undiagnosed comorbidities might be missed. This study is a cross-sectional study that might not show a cause and effect relationship.

## Figures and Tables

**Table 1:** Sociodemographic characteristics of participants in West Gojjam Zone public hospitals, Northwest, Ethiopia, 2021.

Variables	Category	Frequency	Percent (%)
Sex	Male	178	58.6
	Female	126	41.4

Age	<30	33	10.9
	30-49	81	26.6
	50-69	114	37.5
	≥70	76	25.0

Religion	Orthodox	292	96.1
	Others	12	3.9

Marital status	Single	13	4.3
	Married	210	69.1
	Divorced	23	7.6
	Widowed	58	19.1

Residence	Urban	91	29.9
	Rural	213	70.1

Educational level	Unable to read and write	138	45.4
	Only read and write	87	28.6
	Primary school	35	11.5
	High school	30	9.9
	College/university	14	4.6

Occupation	Housewife	25	8.2
	Farmer	204	67.1
	Merchant	32	10.5
	Government employed	17	5.6
	Others	26	8.5

Health insurance	Yes	286	94.1
	No	18	5.9

**Table 2:** Clinical characteristics of HF patients in West Gojjam Zone public hospitals, Northwest, Ethiopia, 2021.

Variables	Category		Frequency (*N*)	Percent (%)
Comorbidity	No		194	63.8
	Yes	HTN	43	14.1
		DM	7	2.3
		KD	31	10.2
		HTN+DM	4	1.3
		HTN+KD	15	4.9
		DM+KD	3	1.0
		Others	7	2.3

NYHA classification	I		7	2.3
	II		97	31.9
	III		160	52.6
	IV		40	13.2

History of hospitalization for the last one year	No		154	50.7
	Yes	One times	107	35.2
		Two times	17	5.6
		Three times	13	4.3
		Four or more times	13	4.3

Duration of illness with HF	<1 year		38	12.5
	≥1 year		266	87.5

^∗^Other comorbidities: asthma, dyspepsia, and blindness. Note: HTN: hypertension; DM: diabetes mellitus; KD: kidney disease.

**Table 3:** Participants' knowledge related to heart failure among heart failure patients in West Gojjam Zone public hospitals, Northwest, Ethiopia, 2021 (*N* = 304).

No.	Variable	Frequency (*N* = 304)			Correct answer	Percent (%)
		Yes	No	I do not know		
	General knowledge questions					
(1)	Exchange of oxygen and carbon dioxide occurs in the heart	55 (18.1%)	51 (16.1%)	198 (65.1%)	51	16.8%
(2)	HF is a condition in which the heart is not able to pump blood through the body in sufficient amounts	169 (55.6%)	5 (1.6%)	130 (42.8%)	169	55.6%
	Heart failure signs and symptom knowledge questions					
(3)	Difficulty in breathing and shortness of breath are symptoms of HF	287 (94.4%)	9 (3%)	8 (2.6%)	287	94.4%
(4)	One of the symptoms when the lungs become congested with fluid is shortness of breath	240 (78.9%)	10 (3.3%)	54 (17.8%)	240	78.9%
(5)	Some patients with severe HF become breathless when they lie flat and feel much better when they sit up	253 (83.2%)	32 (10.5%)	19 (6.3%)	32	10.5%
(6)	Short-term weight gain is one of the signs of worsening HF	96 (31.6%)	14 (4.6%)	194 (63.8%)	96	31.6%
	Heart failure self-care recommendations and disease condition knowledge questions					
(7)	Overwork and stress sometimes cause HF to get worse	249 (81.9%)	11 (3.6%)	44 (14.6%)	249	81.9%
(8)	Sodium causes water retention	156 (51.3%)	11 (3.6%)	137 (45.1%)	156	51.5%
(9)	Diuretics remove fluids from the body	102 (33.6%)	10 (3.3%)	192 (63.2%)	102	33.6%
(10)	HF patients are discouraged from taking medications without food	242 (79.6%)	32 (10.5%)	30 (9.9%)	32	10.5%
(11)	HF patients had better drink more water than healthy people	41 (13.5%)	123 (40.5%)	140 (46.1%)	123	40.5%
(12)	HF patients had better take a high-salt diet	6 (2%)	287 (94.4%)	11 (3.6%)	287	94.4%
(13)	Smoking is good for patients with HF because it promotes the circulation of blood	4 (1.3%)	283 (93.1%)	17 (5.6%)	283	93.1%
(14)	HF patients should not perform exercise regardless of their severity of HF	55 (18.1%)	66 (21.7%)	183 (60.2%)	66	21.7%
(15)	HF patients had better take a hot bath to promote blood circulation	76 (25%)	111 (36.5%)	117 (38.5%)	111	36.5%

**Table 4:** Depression symptoms on HF patients in West Gojjam Zone public hospitals, Northwest, Ethiopia, 2021 (*N* = 304).

No.	Over the last 2 weeks, how often have you been bothered by any of the following problems?	Frequency (*N* = 304)				Percent (%)
		Not at all	Several days	More than half the day	Nearly every day	
(1)	Little interest or pleasure in doing things	240 (78.9%)	42 (13.8%)	19 (6.3%)	3 (1%)	10%
(2)	Feeling down, depressed, or hopeless	216 (71.1%)	61 (20.1%)	24 (7.9%)	3 (1%)	13%
(3)	Trouble falling or staying asleep or sleeping too much	206 (67.8%)	64 (21.1%)	26 (8.6%)	8 (2.6%)	15%
(4)	Feeling tired or having little energy	187 (61.5%)	68 (22.4%)	43 (14.1%)	6 (2%)	19%
(5)	Poor appetite or overeating	166 (54.6%)	80 (26.3%)	46 (15.1%)	12 (3.9%)	23%
(6)	Feeling bad about yourself or that you are a failure or have let yourself or your family down	184 (60.5%)	73 (24%)	41 (13.5%)	6 (2%)	19%
(7)	Trouble concentrating on things, such as reading the newspaper or watching television	243 (79.9%)	48 (15.8%)	12 (3.9%)	1 (0.3%)	8%
(8)	Moving or speaking so slowly that other people could have noticed or the opposite being so fidgety or restless that you have been moving around a lot more than usual	260 (85.5%)	33 (10.9%)	8 (2.6%)	3 (1%)	6%
(9)	Thoughts that you would be better off dead or of hurting yourself	280 (92.1%)	21 (6.9%)	1 (0.3%)	2 (0.7%)	3%

**Table 5:** Social support of HF patients in West Gojjam Zone public hospitals, Northwest Ethiopia, 2021 (*N* = 304).

No.	Variable	Frequency (*N* = 304)	Percent (%)
(1)	How many people are so close to you that you can count on them if you have serious problems?		
	None	2	0.7%
	1-2	102	33.6%
	3-5	184	60.5%
	≥6	16	5.3%

(2)	How much concern do people show in what you are doing?		
	A lot of concern and interest	166	54.6%
	Some concern and interest	88	28.9%
	Uncertain	30	9.9%
	Little concern and interest	20	6.6%

(3)	How easy can you get practical help from neighbors if you should need it?		
	Very easy	179	58.9%
	Easy	64	21.1%
	Possible	41	13.5%
	Difficult	20	6.6%

**Table 6:** Logistic regression analysis result of factors significantly associated with adherence to heart failure SCRs in multivariate analysis in West Gojjam Zone public hospitals, Ethiopia, 2021.

Variables		Self-care adherence		COR (95% CI)	AOR (95% CI)	*P* value
		Good	Poor			
		*N* = 100	*N* = 204			
Age	<30	18	15	7.9 (3.05, 20.58)	2.73 (0.74, 10.12)	0.134
	30-49	42	39	7.1 (3.21, 15.74)	3.37 (1.14, 9.89)	0.028 ^∗^
	50-69	30	84	2.3 (1.08, 5.17)	2.09 (0.77, 5.73)	0.150
	≥70	10	66	1	1	

Education level	No formal education	43	182	1	1	
	Primary school	24	11	9.2 (4.20, 20.29)	3.22 (1.15, 8.99)	0.026 ^∗^
	High school	22	8	11.6 (4.8, 27.92)	4.17 (1.36, 12.76)	0.012 ^∗^
	College/university	11	3	15.5 (4.15, 58.1)	6.17 (1.22, 31.25)	0.028 ^∗^

Depression symptom	Yes	6	40	1	1	
	No	94	164	3.8 (1.56, 9.35)	6.1 (1.92, 19.37)	0.002 ^∗^

Knowledge	Good knowledge	27	15	4.66 (2.35, 9.26)	4.6 (1.82, 11.86)	0.001 ^∗^
	Poor knowledge	73	189	1	1	

Social support	Poor	7	28	0.3 (0.13, 0.73)	0.56 (0.17, 1.92)	0.360
	Moderate	11	77	0.17 (0.09, 0.35)	0.28 (0.12, 0.64)	0.003 ^∗^
	Strong	82	99	1	1	

Note:  ^∗^*P* value < 0.05 (significant association).

## Data Availability

The datasets used and/or analyzed during for this study will be available from the corresponding author without restriction.

## Abbreviations

AOR: Adjusted odds ratio
CI: Confidence interval
DM: Diabetes mellitus
HF: Heart failure
HTN: Hypertension
KD: Kidney disease
NYHA: New York Heart Association
OSS: Oslo Social Support
PHQ: Patient Health Questionnaire
SCR: Self-care recommendations
SPSS: Statistical Package for Social Sciences.

## Supplementary Materials

Supplementary 1 Supplemental File 1: individual self-care behaviors among HF patients, in West Gojjam Zone public hospitals, Northwest, Ethiopia, 2021.

Supplementary 2 Supplemental File 2: binary logistic regression analysis of factors associated with adherence to self-care recommendation among HF patients, in West Gojjam Zone hospitals, Ethiopia, 2021.
