# Supplementary material for: Adherence to Self-Care Recommendations and Associated Factors among Adult Heart Failure Patients in West Gojjam Zone Public Hospitals, Northwest Ethiopia
Source: Int J Chronic Dis. 2022 Dec 21;2022:9673653. doi: 10.1155/2022/9673653 (PMC9798104; doi:10.1155/2022/9673653)
Supplement: Supplementary 1 — Supplemental File 1: individual self-care behaviors among HF patients, in West Gojjam Zone public hospitals, Northwest, Ethiopia, 2021. [file 9673653.f1.docx]

**Supplemental file 1:** Individual self-care behaviors among HF patients, in west Gojjam zone public hospitals, Northwest, Ethiopia, 2021 (**n**=304)

| **No** | **Variables** | **Frequency (N=304)** | | | | | | | | | | | | | | | **Percent (%)** | | |
| --- | --- | --- | --- | --- | --- | --- | --- | --- | --- | --- | --- | --- | --- | --- | --- | --- | --- | --- | --- |
|  |  | **None of the time** | **A little of the time** | | **Some of the time** | | | **A good bit of the time** | | | **Most of the time** | | | **All of the time** | | |  |  |  |
| **Seeking and securing appropriate medical assistance for their HF** | | | | | | | | | | | | | | | | | | | |
|  | I contact my doctor when I see my feet, ankles, legs, or stomach swell. | 0 | 0 | | | 4  (1.3%) | | | 51  (16.8%) | | | 215  (70.7%) | | | 34  (11.2%) | | | 78% | |
|  | I contact my doctor when I feel more short of breath. | 0 | 0 | | | 4  (1.3%) | | | 87  (28.6%) | | | 164  (53.9%) | | | 49  (16.1%) | | | 77% | |
|  | I contact my doctor when I have nausea or do not feel like eating. | 0 | 2  (0.7%) | | | 9  (3%) | | | 102  (33.6%) | | | 146  (48%) | | | 45  (14.8%) | | | 75% | |
|  | I contact my doctor when I realize I am feeling tired all the time. | 0 | 0 | | | 14  (4.6%) | | | 105  (34.5%) | | | 135  (44.4%) | | | 50  (16.4%) | | | 75% | |
|  | I keep my appointments with my doctor. | 0 | 0 | | | 5  (1.6%) | | | 15  (4.9%) | | | 118  (38.9%) | | | 166  (54.6%) | | | 89% | |
| **Being aware of and attending to the effects and results of HF** | | | | | | | | | | | | | | | | | | | |
|  | When I am short of breath, I rest. | 0 | | 0 | | | 12  (3.9%) | | | 107  (35.2%) | | | 160  (52.6%) | | | 25  (8.2%) | | | 73% |
|  | When I am short of breath or tired, I ask for help with something I am unable to do. | 0 | | 3  (1%) | | | 37  (12.2%) | | | 162  (53.3%) | | | 92  (30.3%) | | | 10  (3.3%) | | | 65% |
|  | To help reduce my symptoms, like fatigue or shortness of breath, I limit the activities that are hard for me. | 0 | | 3  (1%) | | | 45  (14.8%) | | | 164  (53.9%) | | | 83  (27.3%) | | | 9  (3%) | | | 63% |
|  | I spread my activities out over the whole day so I do not get too tired. | 0 | | 14  (4.6%) | | | 139  (45.8%) | | | 108  (35.5%) | | | 33  (10.9%) | | | 10  (3.3%) | | | 53% |
|  | I plan rest times during my day. | 5  (1.6%) | | 21  (6.9%) | | | 141  (46.4%) | | | 96  (31.6%) | | | 34  (11.2%) | | | 7  (2.3%) | | | 50% |
| **Effectively carrying out medically prescribed diagnostic, therapeutic, and rehabilitative measures directed toward prevention of exacerbations or complications of HF** | | | | | | | | | | | | | | | | | | | |
|  | I watch how much water I pass (urinate) each day. | 215(70.7%) | | 38(12.5%) | | | 23(7.6%) | | | 18(5.9%) | | | 6(2%) | | | 4(1.3%) | | | 12% |
|  | I am careful not to drink “too many” fluids. | 106(34.9%) | | 77(25.3%) | | | 59(19.4%) | | | 44(14.5%) | | | 12(3.9%) | | | 6(2%) | | | 27% |
|  | I do not eat canned soups. | 3(1%) | | 1(0.3%) | | | 4(1.3%) | | | 36(11.8%) | | | 114(37.5%) | | | 146(48%) | | | 86% |
|  | I take my pills every day. | 0 | | 0 | | | 0 | | | 14(4.6%) | | | 123(40.5%) | | | 167(54.7%) | | | 90% |
|  | I always refill prescriptions for my pills on time. | 2(0.7%) | | 1(0.3%) | | | 0 | | | 25(8.2%) | | | 124(40.8%) | | | 15250% | | | 88% |
|  | I take my pills as the doctor prescribed—I take all the doses of my pills. | 0 | | 0 | | | 0 | | | 24(7.9%) | | | 130(42.8%) | | | 150(49.3%) | | | 88% |
|  | I have a system to help tell me when to take my pills. | 0 | | 1(0.3%) | | | 3(1%) | | | 52(17.1%) | | | 126(41.4%) | | | 122(40.1%) | | | 84% |
|  | I stay away from people who have a cold or flu. | 3(1%) | | 3(1%) | | | 14(4.6%) | | | 130(42.8%) | | | 115(37.8%) | | | 39(12.8%) | | | 71% |
|  | I limit my alcohol intake to 1 glass of beer or wine or 1 shot a day. | 0 | | 0 | | | 2(0.7%) | | | 15(4.9%) | | | 52(17.1%) | | | 235(77.3%) | | | 94% |
|  | I am physically active (e.g., walk or ride a bike) 3 to 4 days per week. | 0 | | 1(0.3%) | | | 5(1.6%) | | | 35(11.5%) | | | 181(59.5%) | | | 82(27%) | | | 82% |
| **Modifying the self-concept in accepting oneself as having HF** | | | | | | | | | | | | | | | | | | | |
|  | I believe that having HF is a condition to which I can adjust. | 0 | | 1(0.3%) | | | 4(1.3%) | | | 54(17.8%) | | | 189(62.2%) | | | 56(18.4%) | | | 79% |
|  | I think a person can live a happy and good life, even after having HF. | 0 | | 1  (0.3%) | | | 23(7.6%) | | | 143(47%) | | | 109(35.9%) | | | 28(9.2%) | | | 69% |
| **Learning to live with the effects of HF and the effects of HF treatments** | | | | | | | | | | | | | | | | | | | |
|  | When I feel anxious about my worsening symptoms of HF, I talk with my doctor about it. | 0 | | 0 | | | 7(2.3%) | | | 56(18.4%) | | | 121(39.8%) | | | 120(39.5%) | | | 83% |
|  | I talk to my doctor and family about my condition to make choices and plans for the future. | 0 | | 1(0.3%) | | | 3(1%) | | | 37(12.2%) | | | 119(39.1%) | | | 144(47.4%) | | | 86% |
|  | I am a nonsmoker. | 0 | | 0 | | | 1(0.3%) | | | 0 | | | 16(5.3%) | | | 287(94.4%) | | | 99% |
|  | I put my feet up when I sit in a chair. | 78(25.7% | | 60(19.7% | | | 107(35.2%) | | | 43(14.1%) | | | 14(4.6%) | | | 2(0.7%) | | | 31% |
