# Supplementary material for: Adherence to Self-Care Recommendations and Associated Factors among Adult Heart Failure Patients in West Gojjam Zone Public Hospitals, Northwest Ethiopia
Source: Int J Chronic Dis. 2022 Dec 21;2022:9673653. doi: 10.1155/2022/9673653 (PMC9798104; doi:10.1155/2022/9673653)
Supplement: Supplementary 2 — Supplemental File 2: binary logistic regression analysis of factors associated with adherence to self-care recommendation among HF patients, in West Gojjam Zone hospitals, Ethiopia, 2021. [file 9673653.f2.docx]

Supplemental file 2: Binary logistic regression analysis of factors associated with adherence to self-care recommendation among HF patients, in west Gojjam zone hospitals, Ethiopia, 2021

| Variable Categories | | Self-Care Adherence | | COR (95% CI) | AOR (95%CI) | P-value |
| --- | --- | --- | --- | --- | --- | --- |
|  | | Good | Poor |  |  |  |
| Age | Less than 30 | 18 (54.5%) | 15 (45.5%) | 7.9  (3.05-20.58) | 2.73  (0.74-10.12) | 0.134 |
|  | 30- 49 | 42 (51.9%) | 39  (48.1%) | 7.1  (3.21-15.74) | 3.37  (1.14-9.89) | **0.028^*^** |
|  | 50- 69 | 30 (26.3%) | 84 (73.7%) | 2.3  (1.08-5.17) | 2.09  (0.77-5.73) | 0.150 |
|  | > 70 | 10 (13.2%) | 66 (86.8%) | 1 | 1 |  |
| Residence | Urban | 52 (57.1%) | 39 (42.9%) | 4.6  (2.71-7.75) | 1.87  (0.87- 4.02) | 0.110 |
|  | Rural | 48 (22.5%) | 165 (77.5%) | 1 | 1 |  |
| Admission | Yes | 35  (23.3%) | 115  (76.7%) | 1 | 1 |  |
|  | No | 65  (42.2%) | 89 (57.8%) | 2.4  (1.46-3.94) | 1.57  (0.7-3.12) | 0.199. |
| Comorbidity | Yes | 25  (22.7%) | 85  (77.3%) | 1 | 1 |  |
|  | No | 75  (38.7%) | 119  (61.3%) | 2.1  (1.27-3.65) | 1.63  (0.76-3.51) | 0.214 |
| NYHA | NYHA III and IV | 47  (23.5%) | 153  (76.5%) | 1 | 1 |  |
|  | NYHA I and II | 53  (51.0%) | 51  (49.0%) | 3.4  (2.04-5.60) | 1.37  (0.68-2.75) | 0.375 |
| Marital status | Married | 79  (37.6%) | 131  (62.4%) | 2.1  (1.199-3.67) | 0.91  (0.43-1.93) | 0.799 |
|  | Single | 21  (22.3%) | 73  (77.7%) | 1 | 1 |  |
| Education Level | No formal Education | 43  (19.1%) | 182  (80.9%) | 1 | 1 |  |
|  | Primary School | 24  (68.6%) | 11  (31.4%) | 9.2  (4.20-20.29) | 3.22  (1.15-8.99) | **0.026^*^** |
|  | High School | 22  (73.3%) | 8  (26.7%) | 11.6  (4.85-27.92) | 4.17  (1.36-12.76) | **0.012^*^** |
|  | College/University | 11  (78.6%) | 3  (21.4%)) | 15.5  (4.15-58.05) | 6.17  (1.22-31.25) | **0.028^*^** |
| Depression symptom | Yes | 6  (13.0%) | 40  (87.0%) | 1 | 1 |  |
|  | No | 94  (36.4%) | 164  (63.6%) | 3.8  (1.56-9.35) | 6.1  (1.92-19.37) | **0.002*** |
| Knowledge | Good knowledge | 27  (64.3%) | 15  (35.7%) | 4.66  (2.35-9.26) | 4.6  (1.82-11.86) | **0.001^*^** |
|  | Poor knowledge | 73  (27.9%) | 189  (72.1%) | 1 | 1 |  |
| Social support | Poor | 7  (20.0%) | 28  (80.0%) | 0.30  (0.13-0.73) | 0.565  (0.17-1.92) | 0.360 |
|  | Moderate | 11  (12.5%) | 77  (87.5%) | 0.17  (0.09-.35) | 0.28  (0.12-0.64) | **0.003^*^** |
|  | Strong | 82  (45.3%) | 99 (54.7%) | 1 | 1 |  |

**^*^P-value <0.05 showing statistically significant association**
